# Detection of Equine Parvovirus-Hepatitis Virus and Equine Hepacivirus in Archived Sera from Horses in France and Australia

**DOI:** 10.3390/v16060862

**Published:** 2024-05-28

**Authors:** Christine Fortier, Charles El-Hage, Camille Normand, Erika S. Hue, Gabrielle Sutton, Christel Marcillaud-Pitel, Kim Jeffers, Nicholas Bamford, Elise Oden, Romain Paillot, Carol Hartley, James Gilkerson, Stéphane Pronost

**Affiliations:** 1 LABÉO, 14280 Saint-Contest, France; christine.fortier@laboratoire-labeo.fr (C.F.); erika.hue@laboratoire-labeo.fr (E.S.H.); gabrielle.sutton.hsj@ssss.gouv.qc.ca (G.S.); elise.oden@laboratoire-labeo.fr (E.O.); romain.paillot@writtle.ac.uk (R.P.); 2 Normandie Université, UNICAEN, Biotargen, 14280 Saint-Contest, France; 3 Centre for Equine Infectious Diseases, Melbourne Veterinary School, The University of Melbourne, Parkville, VIC 3010, Australia; cmeh@unimelb.edu.au (C.E.-H.); kim.jeffers@unimelb.edu.au (K.J.); n.bamford@unimelb.edu.au (N.B.); carolah@unimelb.edu.au (C.H.); jrgilk@unimelb.edu.au (J.G.); 4 Cytokines and Adaptive Immunity Lab, Sainte-Justine University Hospital and Research Center, Université de Montréal, Montréal, QC H3C 3J7, Canada; 5 Microbiology, Infectiology and Immunology Department, Faculty of Medicine, University of Montréal, Montreal, QC H3C 3J7, Canada; 6 RESPE (Réseau d’Epidémio-Surveillance en Pathologie Équine), 14280 Saint-Contest, France; c.marcillaud-pitel@respe.net; 7 Faculty of Science & Engineering, School of Agriculture, Animal & Environmental Sciences, Anglia Ruskin University (ARU Writtle), Lordship Road, Writtle Chelmsford CM1 3RR, UK

**Keywords:** equine parvovirus hepatitis virus, equine hepacivirus, horses, frequency, phylogeny, Australia, France

## Abstract

Reports of newly discovered equine hepatotropic flavi- and parvoviruses have emerged throughout the last decade in many countries, the discovery of which has stimulated a great deal of interest and clinical research. Although commonly detected in horses without signs of disease, equine parvovirus hepatitis (EqPV-H) and equine hepacivirus (EqHV) have been associated with liver disease, including following the administration of contaminated anti-toxin. Our aim was to determine whether EqPV-H and EqHV are present in Australian horses and whether EqPV-H was present in French horses and to examine sequence diversity between strains of both viruses amongst infected horses on either side of the globe. Sera from 188 Australian horses and 256 French horses from horses with and without clinical signs of disease were collected. Twelve out of 256 (4.7%) and 6 out of 188 (3.2%) French and Australian horses, respectively, were positive for the molecular detection of EqPV-H. Five out of 256 (1.9%) and 21 out of 188 (11.2%) French and Australian horses, respectively, were positive for the molecular detection of EqHV. Australian strains for both viruses were genomically clustered, in contrast to strains from French horses, which were more broadly distributed. The findings of this preliminary survey, with the molecular detection of EqHV and EqPV-H in Australia and the latter in France, adds to the growing body of awareness regarding these recently discovered hepatotropic viruses. It has provided valuable information not just in terms of geographic endemicity but will guide equine clinicians, carers, and authorities regarding infectious agents and potential impacts of allogenic tissue contamination. Although we have filled many gaps in the world map regarding equine hepatotropic viruses, further prospective studies in this emerging field may be useful in terms of elucidating risk factors and pathogenesis of these pathogens and management of cases in terms of prevention and diagnosis.

## 1. Introduction

Reports of newly discovered equine hepatotropic flavi- and parvoviruses have emerged throughout the last decade, the discovery of which has stimulated a great deal of interest and clinical research [1,2,3,4]. These viruses have been identified using novel genomic detection techniques, initially on serum products associated with cases of serum hepatitis (Theiler’s disease) in horses. Detected first, the flaviviruses include equine hepacivirus (EqHV), known previously as either non-primate hepacivirus (NPHV) or hepacivirus A [2], and two pegiviruses: equine pegivirus D and Theiler’s disease-associated virus (TDAV)] [3,4,5]. There is no evidence to date to suggest that these pegiviruses are hepatotropic [5,6]. Equine parvovirus-hepatitis (EqPV-H) was more recently reported in 2018 [4], originally in anti-serum associated with cases of Theiler’s disease in recipient horses. The resulting hepatitis was originally considered due to the aforementioned flaviviruses until further studies discovered EqPV-H. The detection of these viruses is not uncommon, with evidence of nucleic acid and/or serological responses to EqHV and EqPV-H in North and South America, Asia, and Europe [6,7,8,9,10,11,12,13,14,15,16]. Updated information on the detection of EqPV-H and EqHV in different countries is presented in Table 1 and Table 2, respectively. To date, they have not been reported in Australia, nor has EqPV-H been reported in France.

Experimental transmission studies have provided evidence that EqPV-H can cause clinicopathological evidence of liver pathology in horses [8,25]. Although experimental studies with EqHV have demonstrated subclinical hepatopathy in horses, the causation of clinical disease has been clouded by the subsequent demonstration of the presence of EqPV-H [8,26]. Both viruses are hepatotropic [4,5,8,26]. These recent findings have been augmented by surveillance studies commonly identifying prolonged viremia in apparently clinically normal horses with EqPV-H and EqHV [1,7,14,16,27,28,29]. Though not all transmission routes have been determined for EqHV and EqPV-H, studies have reported iatrogenic transmission for both [26] and vertical transmission for EqHV [30,31], and horizontal transmission is considered likely for both viruses [8,19,25,30,32].

**Table 2:** EqHV infections in horses detected by country (n.a. = data not available).

Country	References	Number of Sera	qPCR (%)	Serology (%)	PCR Targets	PCR Assay
China	[13]	177	3.4	n.a.	NS5B	Nested PCR
	[33]	133	9	n.a	NS3	Nested PCR
Republic of Korea	[34]	74	18.9	n.a.	NS3	Nested PCR
	[24]	160	8.1	n.a.	NS3	Nested PCR
Germany	[35]	733 ^(1)^	18.3	61.8	5′UTR	Sybr Green assay
	[36]	1158	2.4	n.a.	NS3	equHepaciRT-PCR
Italy	[37]	1932	4.7	n.a.	5′UTR	TaqMan assay
Brazil	[38]	231	13.4	n.a.	5′UTR	TaqMan assay
South Africa	[10]	454 ^(2)^	7.9	83.7	5′UTR	Sybr Green assay
Mongolia	[39]	n.a.	40	n.a.	5′UTR	n.a.
Morocco	[40]	172	10.5	65.7	n.a.	n.a.
France	[16]	1033	6.2	n.a.	5′UTR	TaqMan assay
	This study	256	1.9	n.a.	5′UTR	TaqMan assay
Australia	This study	188	11.2	n.a.	5′UTR	TaqMan assay

Horse populations with no clinical signs of hepatitis; ^(1)^ thoroughbred only; ^(2)^ thoroughbred foals only.

EqHV has been detected in 6.2% of clinically normal French horses, with thoroughbreds being more likely to be infected [16]. In addition to surveillance of the French horses, we hypothesized that opportunistic sampling of Australian horses may indicate the presence of these viruses. Although Theiler’s disease has not been reported in Australian horses [41], it was considered that these viruses are likely to be present, given reports of silent clinical infections and natural transmission in other countries [8,30].

Our aim was to determine whether EqPV-H and EqHV are present in Australian horses and whether EqPV-H is present in French horses. We also sought to further examine sequence diversity between strains of both viruses amongst infected horses on either side of the globe.

## 2. Materials and Methods

### 2.1. Serum Samples

Archived serum samples stored at −80 °C prior to molecular analysis from 188 Australian horses and 256 French horses collected between 2016 and 2019 were accessed for this study. Samples from French horses were documented via the French Epidemio-Surveillance Network (RESPE). Samples from Australian horses were collected for a variety of disease screening purposes nationwide, mostly from surveillance schemes from clinically normal and horses demonstrating signs of disease. Given the nature of the surveillance schemes, there were limited data available regarding history and detailed signalment; however, details including sex, breed, and age are included for both populations in Table 3.

### 2.2. Nucleic Acid Extraction and Quantitative RT-PCR

Nucleic acid extraction of 140 µL sera from French horses was performed using the QIAmp viral RNA Mini kit (Qiagen, Les Ullis, France) according to the manufacturer’s instructions and eluted in a final volume of 50 µL. Australian sera were extracted using the MagMAX™ CORE Nucleic Acid Purification Kit (Thermo Fisher Scientific, Waltham, MA, USA) according to the manufacturer’s instructions with 200 µL serum eluted into in a 90 µL final volume.

All sera were screened for genomic evidence of equine hepacivirus (EqHV) and equine parvovirus-hepatitis (EqPV-H). The EqHV RT-qPCR was carried out using the One-Step Prime Script RT-PCR kit (Takara, Ozyme, Saint-Cyr-l’Ecole, France) and performed as previously described [1] with some adaptations [16]. The EqPV-H qPCR was performed as previously published [16,17] using the Eurobio probe qPCR Mix Low Rox (Eurobio Scientific, Les Ulis, France). Both PCR assays were developed and validated following the AFNOR norm NF-U47-600-2 (NF U 47-600-2, 2015) [42]. The limit of detection was 3.5 × 10^2^ genome copies/mL of serum for EqHV, and the range of quantification was between 3.5 × 10^4^ and 3.5 × 10^9^ genome copies/mL of serum. For EqPV-H, the limit of detection was 3.2 × 10^4^ genome copies/mL of serum, and the range of quantification was between 1.8 × 10^5^ and 1.8 × 10^9^ genome copies/mL of serum.

### 2.3. Sequencing and Phylogenic Analysis

Phylogenetic analysis was performed on an NS5B fragment (308 nt) for EqHV and on an NS1 fragment (587 nt) for EqPV-H.

The NS5B region of EqHV was amplified by nested PCR using primers as described [2], RNA was first reverse-transcribed using the SuperScript III One-Step RT-PCR System with Platinum Taq High Fidelity (Invitrogen, Cergy Pontoise, France) and gene-specific primers. A second PCR was performed using Phusion Hot Start II DNA polymerase (Fisher Scientific, Illkirch-Graffenstaden, France) and inner primers. Sanger sequencing was performed on amplicons using a primer walking strategy.

The NS1 fragments were amplified by PCR using primers as described [14]. This PCR was conducted using Phusion Hot Start II DNA polymerase (Fisher Scientific, Illkirch-Graffenstaden, France). Sanger sequencing was performed on amplicons using a primer walking strategy.

The genomic sequences of NS5B and NS1 reported in this study were submitted to GenBank under the accession numbers shown in Supplementary Tables S1 and S2.

Median-joining networks were built both for EqHV and EqPV-H using Population Analysis with Reticulate Trees (PopART) software v1.7 [43].

### 2.4. Statistics

Binomial logistic regression methods were used to determine significance of host factors, including age, breed, sex, and location in relation to the EqHV and EqPV-H status for Australian horses.

Fisher’s exact test was applied for simple comparison of proportions. Statistical significance was set at *p* < 0.05.

## 3. Results

### 3.1. Study Population

Details of the study populations are outlined in Table 3, where the majority (139/188, 74%) of the Australian horses were clinically normal and predominantly TB geldings. This was in contrast to samples from the 256 French horses, all of which had various clinical diseases and were of a greater average age and a more even sex distribution, most of which were predominantly Arab Saddlebreds.

### 3.2. Virus Detection, Quantification

#### 3.2.1. Equine Parvovirus-Hepatitis Virus

Twelve out of 256 (4.7%) and 6 out of 188 (3.2%) French and Australian horses, respectively, were positive for molecular detection of EqPV-H. Only three positive horses (all from France) recorded genome copies above the limit of quantification, with viral loads from 2.3 × 10^5^ to 6.5 × 10^5^ genome copies per ml of serum. All other positive detections (nine French and all six Australian samples) were below levels of quantification (Table 4). Given the data available, we were unable to reliably statistically analyze risk factors for EqP-H for either population.

#### 3.2.2. Equine Hepacivirus

Five out of 256 (1.9%) and 21 out of 188 (11.2%) French and Australian horses, respectively, were positive for molecular detection of EqHV. Twenty-four horses in total recorded genome copies above the limit of quantification, including 19 out of 21 from Australia and all five French horses with viral loads ranging from 6.1 × 10^4^ to 1.4 × 10^8^ genome copies per ml of serum (Table 5). Binomial logistic regression methods were used to determine the significance of independent (predictor) variables for the Australian population (Supplementary Table S3). Australian horses infected with EqHV were determined to be significantly more likely to be younger (*p* = 0.006) and Thoroughbred (*p* = 0.029).

### 3.3. Phylogenetic Study

#### 3.3.1. Equine Parvovirus-Hepatitis Virus

**Figure 1:**
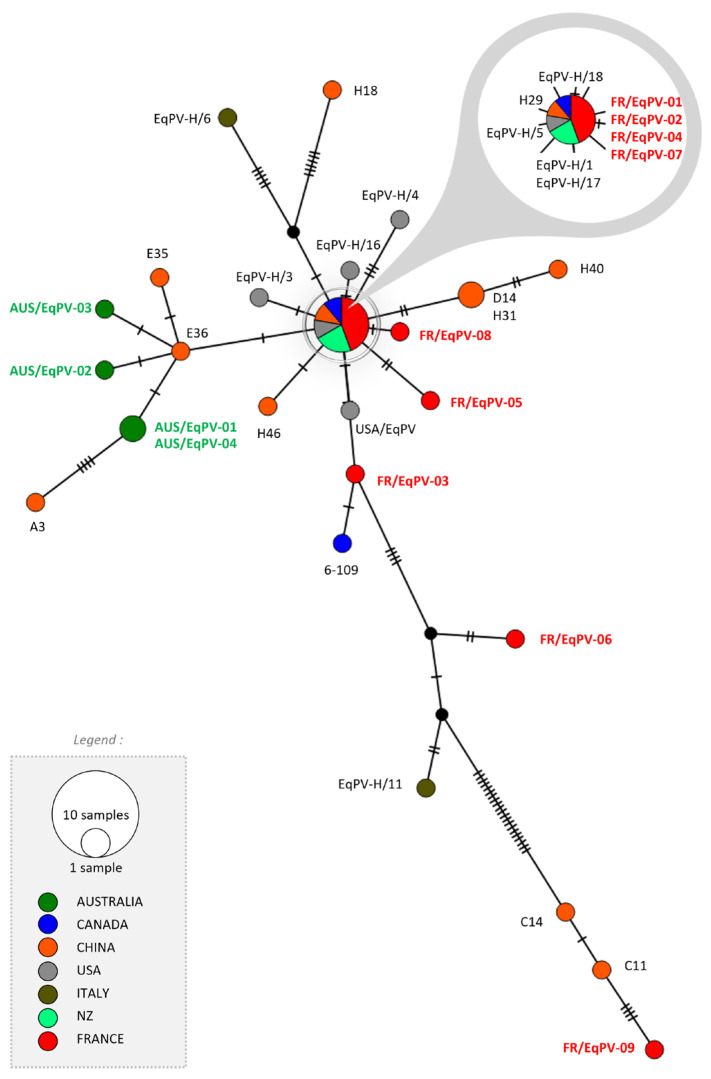
Phylogenetic analysis of parvovirus strains. Network built with 453 nt sequences from NS1. Sequencing of the NS1 fragment of Equine Parvovirus Hepatitis virus based upon country of isolation (color) and size for numbers of samples see insert. Australian and French samples analyzed in this study are presented in green and red, respectively.

The sequencing of the NS1 fragment of EqPV-H (four Australian and nine French strains) demonstrated that the distribution of Australian strains was confined to one cluster, compared to the greater heterogeneity of distribution among French strains. Australian strains were closely related to Chinese strains (Figure 1). All EqPV-H strains presented for the phylogenetic analysis are listed in Supplementary Table S1.

#### 3.3.2. Equine Hepacivirus

**Figure 2:**
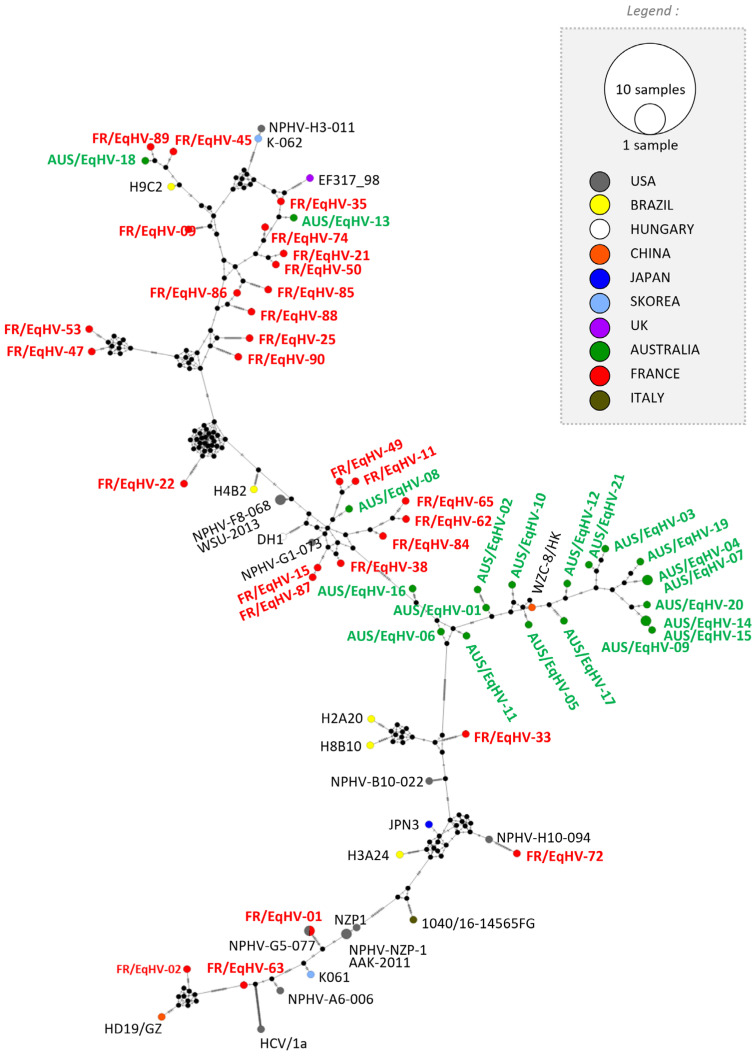
Phylogenetic analysis of hepacivirus strains. Network built with 260 nt sequences from NS5B. Sequencing of the NS5B fragment of hepacivirus based upon country of isolation (color) and size for numbers of samples see insert. Australian and French samples analyzed in this study are presented in green and red, respectively.

The hepacivirus network, obtained after NS5B sequencing (21 Australian strains and 5 French strains), demonstrated heterogeneity in the distribution of French EqHV strains compared to those from Australia. This is illustrated by the cluster Aus formed by 18 Australian strains and one Chinese strain. Nevertheless, three Australian strains are out of the cluster (Figure 2). All strains presented for phylogenetic analysis are listed in Supplementary Table S2. 

## 4. Discussion

To our knowledge, this is the first reported genomic detection of EqHV and EqPV-H in Australian horse populations and the first of the latter virus in French horses. Given the categorical nature of this preliminary survey and opportunistic sampling, the focus was on detection, with limited clinical and epidemiological interpretation possible. Obviously, more extensive data would be valuable to assess putative risk factors, and further detailed studies are required to determine the association of disease with the detection and quantification of these viruses. The overall frequency of genomic detection of both viruses in horse sera from France and Australia remained within previously reported ranges [6,44]. 

Amongst the Australian horses infected with EqHV, despite thoroughbreds being more numerous than other breeds, they remained significantly more likely to be infected compared to other breeds. This finding is consistent with previous reports of several European studies that showed that EqHV detection was relatively higher in thoroughbreds [16,27,35]. 

Racehorses in training may offer an increased opportunity for horizontal transmission when horses in close proximity are handled and mixed as part of a training group. This facilitates greater potential cross-contamination than horses on pasture or those managed more extensively. It remains to be determined whether these management practices or other factors including genetic susceptibility may contribute to the higher prevalence in Thoroughbreds [16,27].

The detection rate in the French population (1.9%) was lower than previously described [16]. As both cases were opportunistic samples, it should be noted that the different characteristics of the two populations may be part of the explanation. In the 2016 retrospective study, we analyzed sera collected over several years (2007 to 2015), 50% of which were from Thoroughbreds. In this study of French samples, all sera were collected in the same year (2016) and Thoroughbreds only constituted 6% of the study population (Table 3). It was noted that the vast majority of positive detections for both viruses in Australian horses were from horses without clinical signs of disease (Table 4 and Table 5). Although this finding may support the subclinical nature of infection, it further emphasizes that more extensive studies are needed to determine epidemiological factors associated with disease following the infection of horses with EqHV and EqPV-H. This observation was not possible in the French population, as all samples were from disease surveillance programs (Table 3).

Quantification of EqPV-H loads may provide a greater opportunity for interpretation of clinical significance; however, this was only possible in 3 out of 12 French horses with a range of 2.3 × 10^5^ to 6.5 × 10^5^ genome copies per ml. These loads are consistent with those described in Germany in Thoroughbreds without clinical signs of disease [14]. Fifteen other horses (nine from France and all six from Australia) were positive; however, levels were below the level of quantification (Table 4).

All five French horses positive for EqHV and 19/21 Australian horses recorded genome copies above the limit of quantification. Viral loads ranged from 6.1 ×10^4^ to 8.5 ×10^7^ and from 3.4 × 10^6^ to 1.4 × 10^8^ copies/mL, respectively. These relatively high viral loads were in agreement with the results of other studies (Table 5).

The standardization of methods was facilitated by simultaneous testing within the same laboratory and time period following similar storage conditions. We remain confident that any discrepancies observed between viral genomic detection from Australian and French samples were considered to be real and not artifact.

Australian strains for both viruses were clustered, in contrast to strains from French horses, which were more broadly distributed. This may reflect exposure to larger numbers of horses within Europe compared to a relatively isolated population in Australia. Australian strains were closely related to Chinese strains while French strains were distributed everywhere except the Australian cluster. Geographically and logistically for Australian horses, there is greater exposure to the Asia-Pacific population of horses that may be reflected in this clustering of strains. While two subtypes of EqHV were reported by our network in a previous French study [16], all strains detected in this study belonged to the NS5B subtype 1, previously identified as the main subtype. This may reflect the relatively smaller number of positive samples and sampling strategy limiting the range of viruses detected.

The detection of EqPV-H and EqHV in French and Australian horses broadens the range of aetiologic agents of hepatopathies to be considered in both regions, offering equine practitioners broader differential diagnostic options for hepatic disease in these countries. A limitation of this study was the lack of correlation between hepatic disease and or associated hematological and or biochemical parameters with virus detection. Although there have been previous reports of hepatic disease of unknown etiology in Australian racehorses and undetermined causes of elevations in hepatic enzymes [45], studies to date have failed to correlate subclinical EqPV-H and EqHV infection with raised liver enzymes [6,22,46]. Similarly, there has been no reported association of liver-associated plasma enzymes to horses with active EqPV-H infection [11,47]. Acute hepatic disease has been well documented following the administration of EqPV-H-contaminated serum; however, many infections remain subclinical. Hence, the pathogenic significance of these viruses and the risk factors associated with disease remains yet to be determined.

Both EqHV and EqPV-H have been detected in a variety of horse populations and are likely endemic in all continents with resident horse populations. It is also worth noting that undocumented routes of transmission for these hepatotropic viruses are likely. Despite detection in sera from diverse types and populations of Australian and French horses, and paucity of reported Theiler’s disease in both countries, continued epidemiological studies are required. Vertical transmission was reported as a likely source of EqHV infection in fetuses in a previous study [30].

The findings of this preliminary survey with molecular detection of EqHV and EqPV-H in Australia, and the latter in France, add to the growing body of awareness regarding these recently discovered hepatotropic viruses. It has provided valuable information not just in terms of geographic endemicity but will guide equine clinicians, carers, and authorities regarding infectious agents and the potential impacts of allogenic tissue contamination.

Although gaps have been filled in the world map regarding the distribution of equine hepatotropic viruses, further prospective studies in this emerging field may be useful in terms of elucidating the risk factors and pathogenesis of these pathogens and the management of cases in terms of prevention and diagnosis.

## Figures and Tables

**Table 1:** EqPV-H infections in horses detected by country (n.a. = data not available).

Country	References	Clinical Status	Nature of Samples	Number of Horses	Pos qPCR (%)	Viral loads (Copies/mL)	Serology (%)	PCR Targets	qPCR Assays
USA	[4]	Theiler’s disease	serum /liver	1	100	n.a.	100	Unbiased amplification and high-throughput sequencing	
		Experimental infection	serum	2	100	approx.10^7^	100	n.a.	TaqMan assay
		No clinical signs	serum	100	13	n.a.	15	VP/NS	Nested PCRs
	[17]	Theiler’s disease	serum	10	90	n.a.	n.a.	VP	TaqMan assay
		In-contact		37	54	n.a.	n.a.		
	[18]	Neurological signs	serum	13	15	n.a.	n.a.	VP	TaqMan assay
		Respiratory signs	serum	14	15	n.a.	n.a.		
		No clinical signs	serum	41	17	n.a.	n.a.		
	[19]	No clinical signs	serum	87	5.7	n.a.	n.a.	Capsid protein ^(2)^	TaqMan assay
			nasal fluid	87	1.1				
		Respiratory clinical signs	serum	667 ^(1)^	6.9				
			nasal fluid	667 ^(1)^	1.2				
China	[7]	No clinical signs	serum	143 (racehorses)	11.9	n.a.	n.a.	VP/NS	Nested PCRs
	[20]	No clinical signs	serum	60 (racehorses)	8.3	n.a.	n.a.	VP	Nested PCR
Germany	[14]	No clinical signs	serum	392 (thoroughbreds)	7.14	1.75 × 10^2^ to 1.25 × 10^5^	34.7	VP	TaqMan assay
Canada	[11]	Theiler’s disease/ in-contact	serum	51	47.1	3.75 × 10^3^ to 3.64 × 10^7^	n.a.	VP	TaqMan assay
Slovenia	[21]	Theiler’s disease	liver	4	100	1.26 × 10^4^ to 2.04 × 10^9 (3)^	n.a.	VP	TaqMan assay
Brazil	[22]	No clinical signs	serum	96	12.5	n.a.	n.a.	NS	Nested PCR
Republic of Korea	[23]	No clinical signs	serum	321	4.4	n.a.	n.a.	NS	Nested PCR
	[24]	^(4)^	serum	160	10.6	n.a.	n.a.	NS	Nested PCR
			fecal samples	114	5.3				
Austria	[9]	No clinical signs	serum	259	8.9	n.a.	30.1	NS	Nested PCR
France	This study	No clinical signs of hepatitis	serum	256	4.7	2.3 × 10^2^ to 6.5 × 10^2^	n.a.	VP	TaqMan assay
Australia	This study	No clinical signs of hepatitis	serum	188	3.2	NQ ^(5)^	n.a.	VP	TaqMan assay

VP: virion protein (capsid protein); NS: non-structural protein; ^(1)^ Including 12 donkeys and 3 mules; ^(2)^ Designed in Pusterla’s study; ^(3)^ GE/million cells; ^(4)^ A total of 253 horses presented for medical treatment with clinical signs (*n* = 107), regular physical examination without clinical signs (*n* = 90) and blood doping test before racing (*n* = 56); ^(5)^ NQ: positive horses below the limit of quantification.

**Table 3:** Details of horses from which archived sera were tested for equine hepacivirus and equine parvovirus-hepatitis from France and Australia.

	France	Australia
Number of horses	256	188
Clinical status NAD	0	139
Clinical status Disease signs	256	49
Age (Average)	0 *–31 (10)	1–22 (6.5)
Male **	132	115
Female	122	73
Unknown sex	2	0
Thoroughbred	22	125
Saddlebred, Arab	113	20
Warmblood	0	10
Pony	28	5
Standardbred	27	0
Unspecified	41	0
Others	25	28

NAD: no abnormalities detected. Disease signs included those consistent with abortion or neurological or respiratory (or pyrexia in French population only); * Youngest horse was 15 days old; ** All males in Australian population and 84/132 in French population were geldings.

**Table 4:** Details of horses testing positive for molecular detection of EqPV-H.

	France	Australia
Number of positive horses	12	6
>LQ (viral load)	3 (2.3 × 10^5^–6.5 × 10^5^ copies/mL)	0
NQ	9	6
Clinical status NAD	0	5
Clinical status disease signs	2 abortions	
	2 neurological	1 respiratory
	8 isolated pyrexia	
Age (Average)	1–24 (10)	4–20 (7.5)
Male	6	3
Female	6	3
Thoroughbred	3	5
Saddlebred, Arab	4	1
Standardbred	2	0
Others	1	0
Unspecified	2	0

NAD: no abnormalities detected. PCR limit of detection is 3.2 × 10^4^ genome copies/mL of serum and limit of quantification (LQ) is 1.8 × 10^5^ genome copies/mL of serum. Positive horses below this value are not quantifiable (NQ).

**Table 5:** Details of horses testing positive for molecular detection of EqHV.

	France	Australia
Number of positive horses	5	21
>LQ (viral load range)	5 (6.1 × 10^4^–8.5 × 10^7^ copies/mL)	19 (3.4 × 10^6^–1.4 × 10^8^ copies/mL)
NQ	0	2
Clinical status NAD	0	20
Clinical status disease signs	1 neurological	1 neurological
	4 isolated pyrexia	
Age (Average)	5–18 (8)	2–7 (4.6)
Male	5	15
Female	0	6
Thoroughbred	1	19
Saddlebred, Arab	2	2
Standardbred	1	0
Unspecified	1	0

NAD: no abnormalities detected. PCR limit of detection is 3.5 × 10^2^ genome copies/mL of serum. and PCR limit of quantification (LQ) is 3.5 × 10^4^ genome copies/mL of serum. Positive horses below this value are not quantifiable (NQ).

## Data Availability

The sequence data are available on GenBank (www.ncbi.nlm.nih.gov/GenBank, accessed on 27 March 2024) under accession numbers PP544270 to PP544308.

## Supplementary Materials

The following supporting information can be downloaded at: https://www.mdpi.com/article/10.3390/v16060862/s1, Table S1: Details of Equine Parvovirus-hepatitis virus strains from this study (Figure 1 in text) and previously reported and lodged with Genbank [4,7,11,14]; Table S2: Details of Equine Hepacivirus strains from this study (Figure 2 in text) and previously reported and lodged with Genbank [2,13,16,28,34,37,48,49,50]; Table S3: Results of univariable logistic regression analysis for the detection of equine hepacivirus (EqHV) from archived sera in Australian horses.
