# Supplementary material for: Detection of Equine Parvovirus-Hepatitis Virus and Equine Hepacivirus in Archived Sera from Horses in France and Australia
Source: Viruses. 2024 May 28;16(6):862. doi: 10.3390/v16060862 (PMC11209535; doi:10.3390/v16060862)
Supplement: Supplementary file 1 [file viruses-16-00862-s001.zip › supplementary table S1.pdf]

Supplementary Table S1 : Details of Equine Parvovirus-hepatitis virus strains from this study (Figure 1 in text) and previously reported and lodged with Genbank.

Virus ID	Abbreviation used in Network	GenBank Accession Number	Localisation	Reference
Equine parvovirus H isolate 6-109	6-109	MN397829	Canada	[11]
Equine parvovirus H strain C11	C11	MH500787	China	[7]
Equine parvovirus H strain C14	C14	MH500788	China	[7]
Equine parvovirus H strain D14	D14	MH500789	China	[7]
Equine parvovirus H strain A3	A3	MH500790	China	[7]
Equine parvovirus H strain E35	E35	MH500791	China	[7]
Equine parvovirus H strain E36	E36	MH500792	China	[7]
Equine parvovirus H strain BCT-01	USA/EqPV	MG136722	USA	[4]
Equine parvovirus H strain H18	H18	MN218583	China	Lu et al. (ds*)
Equine parvovirus H strain H29	H29	MN218584	China	Lu et al. (ds*)
Equine parvovirus H strain H31	H31	MN218585	China	Lu et al. (ds*)
Equine parvovirus H strain H46	H46	MN218586	China	Lu et al. (ds*)
Equine parvovirus H strain H40	H40	MN218587	China	Lu et al. (ds*)
Equine parvovirus H isolate EqPV-H/6	EqPV-H/6	MK792429	Italy	[14]
Equine parvovirus H isolate EqPV-H/1	EqPV-H/1	MK792430	New Zealand	[14]
Equine parvovirus H isolate EqPV-H/3	EqPV-H/3	MK792431	USA	[14]
Equine parvovirus H isolate EqPV-H/4	EqPV-H/4	MK792432	USA	[14]
Equine parvovirus H isolate EqPV-H/5	EqPV-H/5	MK792433	USA	[14]
Equine parvovirus H isolate EqPV-H/11	EqPV-H/11	MK792434	Italy	[14]
Equine parvovirus H isolate EqPV-H/16	EqPV-H/16	MK792435	USA	[14]
Equine parvovirus H isolate EqPV-H/17	EqPV-H/17	MK792436	New Zealand	[14]
Equine parvovirus H isolate EqPV-H/18	EqPV-H/18	MK792437	Canada	[14]
Equine Parvovirus H isolate FR-Eq01/FR/2016	FR/EqPV-01	PP544296	France	This study
Equine Parvovirus H isolate FR-Eq02/FR/2016	FR/EqPV-02	PP544297	France	This study
Equine Parvovirus H isolate FR-Eq03/FR/2016	FR/EqPV-03	PP544298	France	This study
Equine Parvovirus H isolate FR-Eq04/FR/2016	FR/EqPV-04	PP544299	France	This study
Equine Parvovirus H isolate FR-Eq05/FR/2016	FR/EqPV-05	PP544300	France	This study
Equine Parvovirus H isolate FR-Eq06/FR/2016	FR/EqPV-06	PP544301	France	This study
Equine Parvovirus H isolate FR-Eq07/FR/2016	FR/EqPV-07	PP544302	France	This study
Equine Parvovirus H isolate FR-Eq08/FR/2016	FR/EqPV-08	PP544303	France	This study
Equine Parvovirus H isolate FR-Eq09/FR/2016	FR/EqPV-09	PP544304	France	This study
Equine Parvovirus H isolate AUS-Eq01/AUS/2017	AUS/EqPV-01	PP544305	Australia	This study
Equine Parvovirus H isolate AUS-Eq02/AUS/2018	AUS/EqPV-02	PP544306	Australia	This study
Equine Parvovirus H isolate AUS-Eq03/AUS/2018	AUS/EqPV-03	PP544307	Australia	This study
Equine Parvovirus H isolate AUS-Eq04/AUS/2019	AUS/EqPV-04	PP544308	Australia	This study

ds* : direct submission in GenBank
