# Supplementary material for: Detection of Equine Parvovirus-Hepatitis Virus and Equine Hepacivirus in Archived Sera from Horses in France and Australia
Source: Viruses. 2024 May 28;16(6):862. doi: 10.3390/v16060862 (PMC11209535; doi:10.3390/v16060862)
Supplement: Supplementary file 1 [file viruses-16-00862-s001.zip › supplementary table S2.pdf]

Supplementary Table S2 : Details of Equine Hepacivirus strains from this study (Figure 2 in text) and previously reported and lodged with Genbank.

Virus ID	Abbreviation used in Network	GenBank Accession Number	Localisation	Reference
Canine hepacivirus AAK-2011	AAK-2011	JF744991	USA	Kapoor et al. (ds*)
Hepacivirus AK-2012 isolate NPHV-NZP-1	NPHV-NZP-1	JQ434001	USA	Burbelo et al. (ds*)
Hepacivirus AK-2012 isolate NPHV-G1-073	NPHV-G1-073	JQ434002	USA	Burbelo et al. (ds*)
Hepacivirus AK-2012 isolate NPHV-A6-006	NPHV-A6-006	JQ434003	USA	Burbelo et al. (ds*)
Hepacivirus AK-2012 isolate NPHV-B10-022	NPHV-B10-022	JQ434004	USA	Burbelo et al. (ds*)
Hepacivirus AK-2012 isolate NPHV-F8-068	NPHV-F8-068	JQ434005	USA	Burbelo et al. (ds*)
Hepacivirus AK-2012 isolate NPHV-G5-077	NPHV-G5-077	JQ434006	USA	Burbelo et al. (ds*)
Hepacivirus AK-2012 isolate NPHV-H10-094	NPHV-H10-094	JQ434007	USA	Burbelo et al. (ds*)
Hepacivirus AK-2012 isolate NPHV-H3-011	NPHV-H3-011	JQ434008	USA	Burbelo et al. (ds*)
Equine hepacivirus strain 1040/16-14565FG	1040/16-14565FG	KY695220	Italy	[37]
Hepacivirus horse/DH1/HUN/2013	DH1	KF177391	Hungary	[48]
Equine hepacivirus isolate H2A20	H2A20	KT006293	Brazil	[49]
Equine hepacivirus isolate H3A24	H3A24	KT006294	Brazil	[49]
Equine hepacivirus isolate H4B2	H4B2	KT006295	Brazil	[49]
Equine hepacivirus isolate H8B10	H8B10	KT006299	Brazil	[49]
Equine hepacivirus isolate H9C2	H9C2	KT006300	Brazil	[49]
Equine hepacivirus strain WZC-8/HK/China	WZC-8/HK	KU746991	China	[13]
Equine hepacivirus strain HD19/GZ/China	HD19/GZ	KU746994	China	[13]
Equine hepacivirus JPN3/JAPAN/2013	JPN3	NC_024889	Japan	Moriishi et al. (ds*)
Equine hepacivirus strain K-061	K-061	KX056116	South Korea	[34]
Equine hepacivirus strain K-062	K-062	KX056117	South Korea	[34]
Non-primate hepacivirus NZP1	NZP1	KP325401	USA	[28]
Hepacivirus SMKL-2012 isolate NPHV_EF317_98	EF317_98	JX948119	Scotland	[2]
Equine hepacivirus isolate WSU-2013	WSU-2013	KJ472766	USA	Ramsay et al. (ds*)
Equine hepacivirus isolate FR-Eq01/FR/2013	FR/EqHV-01	KX239410	France	[16]
Equine hepacivirus isolate FR-Eq02/FR/2013	FR/EqHV-02	KX239411	France	[16]
Equine hepacivirus isolate FR-Eq09/FR/2013	FR/EqHV-09	KX239415	France	[16]
Equine hepacivirus isolate FR-Eq11/FR/2013	FR/EqHV-11	KX239416	France	[16]
Equine hepacivirus isolate FR-Eq15/FR/2013	FR/EqHV-15	KX239420	France	[16]
Equine hepacivirus isolate FR-Eq21/FR/2013	FR/EqHV-21	KX239423	France	[16]
Equine hepacivirus isolate FR-Eq22/FR/2013	FR/EqHV-22	KX239424	France	[16]
Equine hepacivirus isolate FR-Eq25/FR/2013	FR/EqHV-25	KX239425	France	[16]
Equine hepacivirus isolate FR-Eq33/FR/2014	FR/EqHV-33	KX239433	France	[16]
Equine hepacivirus isolate FR-Eq35/FR/2014	FR/EqHV-35	KX239435	France	[16]
Equine hepacivirus isolate FR-Eq38/FR/2014	FR/EqHV-38	KX239437	France	[16]
Equine hepacivirus isolate FR-Eq45/FR/2014	FR/EqHV-45	KX239444	France	[16]
Equine hepacivirus isolate FR-Eq47/FR/2014	FR/EqHV-47	KX239446	France	[16]
Equine hepacivirus isolate FR-Eq49/FR/2014	FR/EqHV-49	KX239448	France	[16]
Equine hepacivirus isolate FR-Eq50/FR/2014	FR/EqHV-50	KX239449	France	[16]
Equine hepacivirus isolate FR-Eq53/FR/2014	FR/EqHV-53	KX239452	France	[16]
Equine hepacivirus isolate FR-Eq62/FR/2014	FR/EqHV-62	KX239459	France	[16]
Equine hepacivirus isolate FR-Eq63/FR/2014	FR/EqHV-63	KX239460	France	[16]

Equine hepacivirus isolate FR-Eq65/FR/2014	FR/EqHV-65	KX239462	France	[16]
Equine hepacivirus isolate FR-Eq72 /FR/2015	FR/EqHV-72	MN229480	France	[16]
Equine hepacivirus isolate FR-Eq74 /FR/2015	FR/EqHV-74	MN229482	France	[16]
Equine hepacivirus isolate FR-Eq84 /FR/2015	FR/EqHV-84	MN229483	France	[16]
Equine hepacivirus isolate FR-Eq85 /FR/2015	FR/EqHV-85	MN229484	France	[16]
Hepatitis C virus subtype 1a	HCV/1a	AF009606	USA	[50]
Equine hepacivirus isolate FR-Eq86/FR/2016	FR/EqHV-86	PP544270	France	This study
Equine hepacivirus isolate FR-Eq87/FR/2016	FR/EqHV-87	PP544271	France	This study
Equine hepacivirus isolate FR-Eq88/FR/2016	FR/EqHV-88	PP544272	France	This study
Equine hepacivirus isolate FR-Eq89/FR/2016	FR/EqHV-89	PP544273	France	This study
Equine hepacivirus isolate FR-Eq90/FR/2016	FR/EqHV-90	PP544274	France	This study
Equine hepacivirus isolate AUS-Eq01/AUS/2017	AUS/EqHV-01	PP544275	Australia	This study
Equine hepacivirus isolate AUS-Eq02/AUS/2017	AUS/EqHV-02	PP544276	Australia	This study
Equine hepacivirus isolate AUS-Eq03/AUS/2017	AUS/EqHV-03	PP544277	Australia	This study
Equine hepacivirus isolate AUS-Eq04/AUS/2019	AUS/EqHV-04	PP544278	Australia	This study
Equine hepacivirus isolate AUS-Eq05/AUS/2019	AUS/EqHV-05	PP544279	Australia	This study
Equine hepacivirus isolate AUS-Eq06/AUS/2019	AUS/EqHV-06	PP544280	Australia	This study
Equine hepacivirus isolate AUS-Eq07/AUS/2019	AUS/EqHV-07	PP544281	Australia	This study
Equine hepacivirus isolate AUS-Eq08/AUS/2018	AUS/EqHV-08	PP544282	Australia	This study
Equine hepacivirus isolate AUS-Eq09/AUS/2018	AUS/EqHV-09	PP544283	Australia	This study
Equine hepacivirus isolate AUS-Eq10/AUS/2018	AUS/EqHV-10	PP544284	Australia	This study
Equine hepacivirus isolate AUS-Eq11/AUS/2018	AUS/EqHV-11	PP544285	Australia	This study
Equine hepacivirus isolate AUS-Eq12/AUS/2018	AUS/EqHV-12	PP544286	Australia	This study
Equine hepacivirus isolate AUS-Eq13/AUS/2018	AUS/EqHV-13	PP544287	Australia	This study
Equine hepacivirus isolate AUS-Eq14/AUS/2018	AUS/EqHV-14	PP544288	Australia	This study
Equine hepacivirus isolate AUS-Eq15/AUS/2018	AUS/EqHV-15	PP544289	Australia	This study
Equine hepacivirus isolate AUS-Eq16/AUS/2018	AUS/EqHV-16	PP544290	Australia	This study
Equine hepacivirus isolate AUS-Eq17/AUS/2018	AUS/EqHV-17	PP544291	Australia	This study
Equine hepacivirus isolate AUS-Eq18/AUS/2018	AUS/EqHV-18	PP544292	Australia	This study
Equine hepacivirus isolate AUS-Eq19/AUS/2020	AUS/EqHV-19	PP544293	Australia	This study
Equine hepacivirus isolate AUS-Eq20/AUS/2020	AUS/EqHV-20	PP544294	Australia	This study
Equine hepacivirus isolate AUS-Eq21/AUS/2020	AUS/EqHV-21	PP544295	Australia	This study

ds* : direct submission in GenBank
