# Supplementary material for: Detection of Equine Parvovirus-Hepatitis Virus and Equine Hepacivirus in Archived Sera from Horses in France and Australia
Source: Viruses. 2024 May 28;16(6):862. doi: 10.3390/v16060862 (PMC11209535; doi:10.3390/v16060862)
Supplement: Supplementary file 1 [file viruses-16-00862-s001.zip › supplementary table S3.pdf]

Supplementary Table S3 : Results of a univariable logistic regression analysis for the detection of equine hepacivirus (EqHV) from archived sera in Australian horses.

	**EqHV negative (n=167)**<br>**n (%)**<br>**median (IQR)**	**EqHV positive (n=21)**<br>**n (%)**<br>**median (IQR)**	**Crude OR**	**95% CI**	**P value**
Age (years)	5.5 (5 - 7.25)	4 (3 - 6.5)	0.68	0.52 - 0.89	**0.006**
Sex					
Male	91 (56%)	14 (67%)	Ref.		
Female	71 (44%)	7 (33%)	0.64	0.25 - 1.67	0.36
Breed					
TB	105 (64%)	19 (90%)	Ref.		
Non-TB	58 (36%)	2 (10%)	0.19	0.04 - 0.85	**0.029**
Property					
1	45 (27%)	4 (19%)	Ref.		
2	39 (23%)	12 (57%)	3.46	1.03 - 11.6	**0.044**
3	52 (31%)	3 (14%)	0.65	0.14 - 3.06	0.58
4	31 (19%)	2 (10%)	0.73	0.13 - 4.21	0.72

Multivariable logistic regression model

Variables with $P<0.2$ were evaluated for inclusion in multivariable logistic regression model using a backwards stepwise approach, removing least significant variable each time. Only "age" remained significant at the final step, demonstrating that a multivariable logistic regression model was not applicable in this analysis.
